# The Genome Sequences of Three *Paraburkholderia* sp. Strains Isolated from Wood-Decay Fungi Reveal Them as Novel Species with Antimicrobial Biosynthetic Potential

**DOI:** 10.1128/MRA.00778-19

**Published:** 2019-08-22

**Authors:** Gordon Webster, Alex J. Mullins, Aimee S. Bettridge, Cerith Jones, Edward Cunningham-Oakes, Thomas R. Connor, Julian Parkhill, Eshwar Mahenthiralingam

**Affiliations:** a Microbiomes, Microbes and Informatics Group, Organisms and Environment Division, School of Biosciences, Cardiff University, Cardiff, Wales, United Kingdom; b Wellcome Sanger Institute, Wellcome Genome Campus, Cambridge, United Kingdom; University of Delaware

## Abstract

Three strains of fungus-associated *Burkholderiales* bacteria with antagonistic activity against Gram-negative plant pathogens were genome sequenced to investigate their taxonomic placement and potential for antimicrobial specialized metabolite production. The selected strains were identified as novel taxa belonging to the genus Paraburkholderia and carry multiple biosynthetic gene clusters.

## ANNOUNCEMENT

The genus Paraburkholderia comprises a diverse group of environmental bacteria that live in close association with plant and fungal tissues (1, 2), several species of which have been reclassified from Burkholderia (3, 4). *Burkholderia* species produce a variety of specialized metabolites with antimicrobial properties, allowing them to suppress plant disease and kill microbial pathogens (5–7). Evidence suggests that *Paraburkholderia* species also protect plants from disease and promote plant growth by N_2_ fixation and/or other means (1, 8). Furthermore, analysis of *Burkholderia* genomes has revealed numerous novel biosynthetic gene clusters, offering excellent potential for antibiotic discovery (6). To clarify their taxonomy and potential for biosynthesis of specialized metabolites, we determined the genome sequences and antagonistic bioactivity of three recently isolated *Paraburkholderia* sp. strains (9).

Three *Paraburkholderia* sp. strains (BCC1884, BCC1885, and BCC1886) were isolated from the mycelial cords of Phanerochaete sp. PW271 or decaying beech wood (colonized by Vuilleminia comedens) collected from Whitestone Woods, Monmouthshire, United Kingdom (9). Briefly, washed pieces of mycelial cord or beech were incubated on 2% (wt/vol) malt extract agar plates at 20°C. Once bacteria could be observed tracking fungi, they were subcultured onto fresh medium. Bacteria were purified by streaking several times to obtain single colonies.

**FIG 1:**
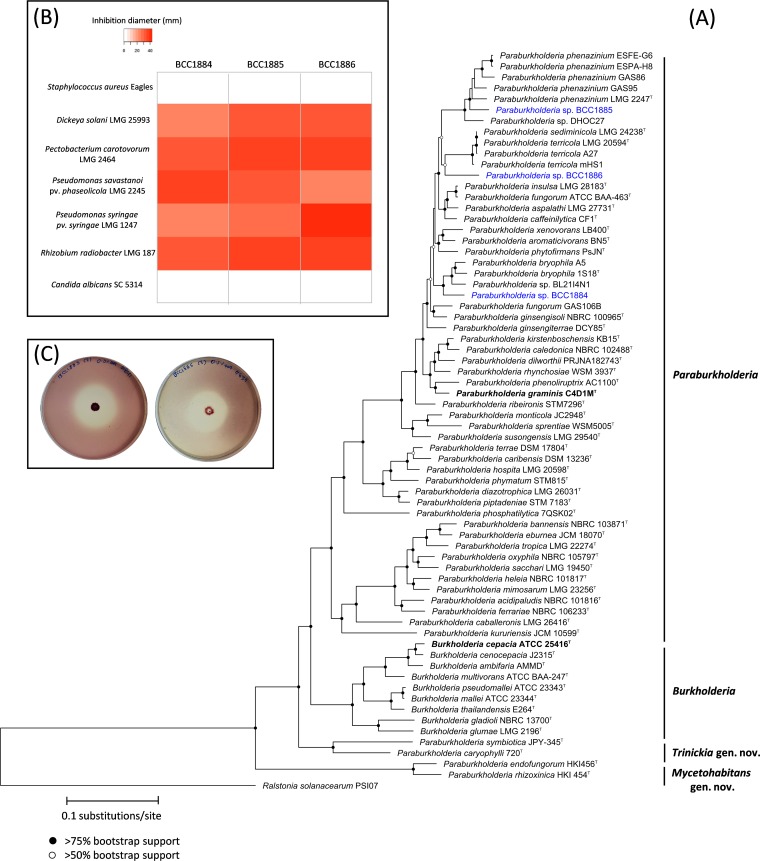
Phylogeny and bioactivity of three *Paraburkholderia* sp. strains (BCC1884, BCC1885, and BCC1886) isolated from wood-decay fungi. (A) rMLST phylogeny of novel *Paraburkholderia* strains within the context of reference *Paraburkholderia* and *Burkholderia* species. The phylogenetic tree was constructed using RAxML v8.2.11 using the maximum likelihood method with the general time-reversible model and gamma distribution. The *Paraburkholderia* strains from this study are shown in blue, and bold black names denote type species for the genera *Burkholderia* and *Paraburkholderia*. New genera are as described by Estrada-de Los Santos et al. (12). Bootstrap support (100 replicates) are shown for support above 50%. (B) Antimicrobial activity heat map of the novel *Paraburkholderia* strains defined by measuring the diameter of the zones of inhibition (mm); *n* = 3 antagonism overlays of each *Paraburkholderia* strain against a panel of human and plant pathogens. The heat map shows the mean zone of inhibition. (C) Examples of antagonism overlay assays; *Paraburkholderia* sp. BCC1885 with Dickeya solani (left) and *Paraburkholderia* sp. BCC1885 with Pectobacterium carotovorum (right). All assays were carried out with 2 μl of an overnight culture of *Paraburkholderia* spotted onto 0.5% (wt/vol) malt extract agar plates, incubated at room temperature for 5 days, and overlaid with a susceptibility organism as previously described (10).

For genome sequencing, strains were grown in 3 ml tryptone soya broth at room temperature for 2 days. The cells were pelleted by centrifugation at 4,000 rpm using an ALC PK120 centrifuge for 10 min, and genomic DNA was extracted using an automated Maxwell 16 instrument with tissue DNA purification kits (Promega) according to the manufacturer’s protocol. Sequencing was performed on an Illumina HiSeq X instrument using a TruSeq DNA library preparation kit. For each genome, 3 to 3.5 million paired reads (150 bp) were generated. All genomes sequenced exceeded 50× coverage. Illumina adaptors were trimmed (TrimGalore v0.4.2), read quality was assessed using FastQC v0.10.1, and contigs were assembled *de novo* with SPAdes v3.9.1 using default settings. The genome sizes and other metrics for the assemblies are as follows: BCC1884, 7.54 Mbp, 62.21% G+C content, 693,480-bp *N*_50_; BCC1885, 8.01 Mbp, 62.36% G+C content, 225,561-bp *N*_50_; and BCC1886, 7.68 Mbp, 62.86% G+C content, 290,160-bp *N*_50_. The *Paraburkholderia* strains were subject to average nucleotide identity (ANI) analysis using PyANI (https://github.com/widdowquinn/pyani), and their phylogenetic relationship to other *Paraburkholderia* species was inferred by ribosomal multilocus sequence typing (rMLST) (Fig. 1A). The rMLST profiles (pubMLST; https://pubmlst.org/) were aligned with MAFFT, and the phylogenetic tree was constructed with RAxML v8.2.11. All neighboring type species of *Paraburkholderia* possessed a genome ANI value below 90%, and each *Paraburkholderia* strain was phylogenetically distinct (Fig. 1A), indicating that all three strains represented novel species.

Bioactivity screening (*n* = 3 per treatment) using an antagonism overlay assay (10) on 0.5% (wt/vol) malt extract medium showed that all strains had anti-Gram-negative activity but no anti-Gram-positive or antifungal activity when screened against a panel of human and plant pathogens (Fig. 1B and C). In summary, bioactivity was observed against Dickeya solani, Pectobacterium carotovorum, Pseudomonas savastanoi, Pseudomonas syringae, and Rhizobium radiobacter. Genome mining with antiSMASH (11) revealed biosynthetic gene clusters for novel bacteriocins, phosphonates, nonribosomal peptides, and polyketides in all three strains. The bioactivity of these strains and functions of the antimicrobial compounds are under investigation.

### Data availability.

The genome sequences and Illumina raw sequence reads have been deposited in the European Nucleotide Archive (ENA) under the ENA project/study number PRJEB31717. The ENA accession numbers for the genome sequences are as follows: CAAJGL010000000 for BCC1884, CAAJGM010000000 for BCC1885, and CAAJGK010000000 for BCC1886; the raw reads are deposited in the NCBI SRA under the accession numbers ERS1333584 (BCC1884), ERS1328826 (BCC1885), and ERS1328912 (BCC1886). The rMLST Burkholderia cepacia complex database numbers 2501, 2502, and 2503 for BCC1884, BCC1885, and BCC1886, respectively, are hosted on the Bacterial Isolate Genome Sequence Database (BIGSdb).
